# Down-regulation of E-cadherin enhances prostate cancer chemoresistance via Notch signaling

**DOI:** 10.1186/s40880-017-0203-x

**Published:** 2017-03-29

**Authors:** Wenchu Wang, Lihui Wang, Atsushi Mizokami, Junlin Shi, Chunlin Zou, Jinlu Dai, Evan T. Keller, Yi Lu, Jian Zhang

**Affiliations:** 1 Center for Translational Medicine, Guangxi Medical University, 12th Floor, Medical Science Research Building, No. 22 Shuangyong Road, Nanning, Guangxi 530021 P. R. China; 2 Key Laboratory of Longevity and Ageing-related Diseases, Ministry of Education, Nanning, Guangxi 530021 P. R. China; 3 Department of Urology and Pathology, School of Medicine, University of Michigan, Ann Arbor, MI 48109 USA; 4 Department of Urology, Graduate School of Medical Sciences, Kanazawa University, Kanazawa, 920-1192 Japan; 5 Department of Biology and School of Medicine, Southern University of Science and Technology, Shenzhen, Guangdong 518055 P. R. China; 6 Department of Urology, University of Pittsburgh School of Medicine, Pittsburgh, PA 15232 USA

**Keywords:** Epithelial-to-mesenchymal transition, E-cadherin, Chemoresistance, Notch signaling, Prostate cancer

## Abstract

**Background:**

The chemoresistance of prostate cancer (PCa) is invariably associated with the aggressiveness and metastasis of this disease. New emerging evidence indicates that the epithelial-to-mesenchymal transition (EMT) may play pivotal roles in the development of chemoresistance and metastasis. As a hallmark of EMT, E-cadherin is suggested to be a key marker in the development of chemoresistance. However, the molecular mechanisms underlying PCa chemoresistance remain unclear. The current study aimed to explore the association between EMT and chemoresistance in PCa as well as whether changing the expression of E-cadherin would affect PCa chemoresistance.

**Methods:**

Parental PC3 and DU145 cells and their chemoresistant PC3-TxR and DU145-TxR cells were analyzed. PC3-TxR and DU145-TxR cells were transfected with E-cadherin-expressing lentivirus to overexpress E-cadherin; PC3 and DU145 cells were transfected with small interfering RNA to silence E-cadherin. Changes of EMT phenotype-related markers and signaling pathways were assessed by Western blotting and quantitative real-time polymerase chain reaction. Tumor cell migration, invasion, and colony formation were then evaluated by wound healing, transwell, and colony formation assays, respectively. The drug sensitivity was evaluated using MTS assay.

**Results:**

Chemoresistant PC3-TxR and DU145-TxR cells exhibited an invasive and metastatic phenotype that associated with EMT, including the down-regulation of E-cadherin and up-regulation of Vimentin, Snail, and N-cadherin, comparing with that of parental PC3 and DU145 cells. When E-cadherin was overexpressed in PC3-TxR and DU145-TxR cells, the expression of Vimentin and Claudin-1 was down-regulated, and tumor cell migration and invasion were inhibited. In particular, the sensitivity to paclitaxel was reactivated in E-cadherin-overexpressing PC3-TxR and DU145-TxR cells. When E-cadherin expression was silenced in parental PC3 and DU145 cells, the expression of Vimentin and Snail was up-regulated, and, particularly, the sensitivity to paclitaxel was decreased. Interestingly, Notch-1 expression was up-regulated in PC3-TxR and DU145-TxR cells, whereas the E-cadherin expression was down-regulated in these cells comparing with their parental cells. The use of γ-secretase inhibitor, a Notch signaling pathway inhibitor, significantly increased the sensitivity of chemoresistant cells to paclitaxel.

**Conclusion:**

The down-regulation of E-cadherin enhances PCa chemoresistance via Notch signaling, and inhibiting the Notch signaling pathway may reverse PCa chemoresistance.

## Background

Prostate cancer (PCa) is the most common cancer and the second leading cause of cancer-related deaths in men in western countries [1, 2]. In 2014, approximately 233,000 new cases of PCa were diagnosed and an estimated 29,480 deaths occurred in the United States [2]. The incidence of PCa in China is increasing as the lifespan has dramatically increased in the Chinese population over the last several decades [3]. Although more than 50% of PCa cases initially respond to androgen-deprivation therapy, most progress to castration-resistant PCa at advanced stages and then become incurable [4–6]. Chemotherapy is a major clinical treatment for castration-resistant PCa. However, multidrug resistance remains a key challenge for the success of chemotherapy [7, 8]. Chemoresistant metastatic PCa is the most lethal form of cancer in adult men [9]. Thus, effective and alternative treatments of PCa are required.

Various steps are involved in cancer progression, including the epithelial-to-mesenchymal transition (EMT). However, whether EMT plays a role in the chemoresistance of PCa remains unclear. EMT is characterized by the down-regulation of E-cadherin, an epithelial marker; the up-regulation of mesenchymal markers, such as Vimentin, N-cadherin, and Snail; and the potently enhanced ability of tumor cell invasion and metastasis [10–13]. The expression of E-cadherin is used to monitor the epithelial phenotype; loss of E-cadherin expression is considered a hallmark of EMT, and reduced E-cadherin expression occurs during the progression of PCa such as migration, invasion, and finally metastasis [14–16]. Recent evidence indicated that EMT not only causes increased invasion and metastasis but also associates with chemoresistance in PCa [17–19]. E-cadherin was suggested to be a key marker in the development of chemoresistance [20, 21]. However, it has not yet been experimentally confirmed whether E-cadherin dominates the process of acquiring chemoresistance.

EMT is triggered by the tightly regulated interplay between signaling pathways, such as the interplay between the Wnt/β-catenin and Akt pathways [22, 23]. Recently, Notch signaling was identified to play a significant role in EMT in different cancers [24, 25]. Notch promotes EMT by regulating several transcriptional and growth factors, including transforming growth factor-β (TGF-β), Snail, and Slug [26–28]. The Notch signaling pathway may also play an important role in cancer chemoresistance. For example, the activity of Notch-1 in tumor tissues is associated with the resistance to tamoxifen in breast cancer patients [29]. In addition, chemotherapy-induced Notch-1 activation is linked to the acquired chemoresistant phenotype of colon cancer cells [30]. Yet, whether the Notch pathway is associated with E-cadherin in the chemoresistance of PCa remains to be determined.

In the current study, we aimed to explore whether EMT, especially the epithelial marker E-cadherin, plays a role in the chemoresistance of PCa and tried to identify new therapeutic targets. We first determined the morphology and functional characteristics of chemoresistant PCa cells. Subsequently, we regulated the expression of E-cadherin to investigate the roles of E-cadherin and its associated signaling pathways in the chemoresistance of PCa cells.

## Methods

### Cell culture and reagents

Human prostate cancer cell lines PC3 and DU145 were obtained from the American Type Culture Collection (Manassas, VA, USA). They were maintained in RPMI-1640 supplemented with 10% fetal bovine serum (FBS) and 1% penicillin and streptomycin (Invitrogen, Carlsbad, CA, USA). The paclitaxel-resistant cell lines PC3-TxR and DU145-TxR were kind gifts provided by Professor Atsushi Mizokami (Kanazawa University, Kanazawa, Japan) and were maintained in 10 nmol/L paclitaxel-contained RPMI-1640 medium. Cell morphology was obsevered under an inverted microscope (Olympus, Tokyo, Japan) at 200× magnification. Paclitaxel was purchased from Invitrogen. Antibodies against E-cadherin, Vimentin, Snail, β-catenin, Claudin-1, Notch-1, Notch-2, Notch-4, Akt, glycogen synthase kinase-3β (GSK-3β), phosphorylated GSK-3β (p-GSK-3β), and nuclear factor kappa-light-chain-enhancer of activated B cells (NF-κB) p65 were purchased from Cell Signaling Technology (Beverly, MA, USA) and β-actin was from Sigma (St. Louis, MO, USA). Human E-cadherin-specific small interfering RNAs (siRNAs), control siRNA, and lentiviral E-cadherin and control vectors were purchased from Genepharma (Shanghai, China). The luciferase reporter lentiviral vector and luciferase-expressing cells were constructed in our own laboratory. The γ-secretase inhibitor (GSI, a Notch inhibitor) was purchased from Calbiochem (San Diego, CA, USA).

### Animal experiments

Five- to 6-week-old, male severe combined immune deficiency (SCID) mice were purchased from Beijing HFK Bioscience (Beijing, China). The animal experiment protocol was approved by the Institutional Animal Care and Use Committee, Guangxi Medical University. All mice were housed under specific pathogen-free conditions in accordance with National Institutes of Health (NIH) guidelines. Ten mice per group were used. PC3-luc and PC3-TxR-luc cells (4 × 10^6^ cells/mouse, mixed with Matrigel [Invitrogen]) were injected subcutaneously as indicated, respectively. Tumor growth was monitored weekly using a bioluminescence imaging system (Bruker, Billerica, MA, USA). Tumor size was measured once a week, and tumor volume was calculated using the following formula: volume = length × width^2^ × 0.52 [31]. Mice were euthanized by neck dislocation when the tumor volume was approximately 1 cm^3^, and tumors were excised and photographed.

### Semi-quantitative reverse transcription-polymerase chain reaction (RT-PCR) and quantitative real-time PCR

Total RNA was extracted from PC3, DU145, PC3-TxR, and DU145-TxR cells using Trizol reagent (Invitrogen) according to the manufacturer’s protocol. Two micrograms of total RNA were used in each reverse transcription. The mRNA expression of E-cadherin, Snail, N-cadherin, Vimentin, E-box binding homeobox-1(ZEB-1), Twist, Smad3, β-catenin, Notch-1, and transforming growth factor-β (TGF-β) was detected. The primers for semi-quantative RT-PCR and quantitative real-time PCR (qPCR) are listed in Table 1. qPCR was performed using a SYBR green assay system on an ABI 7300 machine (Applied Biosystems, Waltham, MA, USA) as described previously [32]. Triplicate reactions were performed for each cDNA sample. Data of each gene were confirmed using biological replicate samples. The relative expression of each gene to glyceraldehyde 3-phosphate dehydrogenase (*GAPDH*) was calculated using the ΔCT method [33].

**Table 1:** Primers for semi-quantitative reverse transcription-polymerase chain reaction (RT-PCR) and quantitative real-time PCR

Name	Forward sequence	Reverse sequence	Product length (bp)
E-cadherin	5′-CGGGAATGCAGTTGAGGATC-3′	5′-AGGATGGTGTAAGCGATGGC-3′	201
Snail	5′-GAAAGGCCTTCAACTGCAAA-3′	5′-TGACATCTGAGTGGGTCTGG-3′	249
N-cadherin	5′-AGCCTGGAACATATGTGATGA-3′	5′-CCATAAAACGTCATGGCAGTAA-3′	325
Vimentin	5′-GACAATGCGTCTCTGGCACGTCTT-3′	5′-TCCTCCGCCTCCTGCAGGTTCTT-3′	236
ZEB-1	5′-TTCAAACCCATAGTGGTTGCT-3′	5′-TGGGAGATACCAAACCAACTG-3′	151
Twist	5′-GGAGTCCGCAGTCTTACGAG-3′	5′-TCTGGAGGACCTGGTAGAGG-3′	201
Smad3	5′-ACCAGGGCTTTGAGGCTGTC-3′	5′-GCAAAGGCCCATTCAGGTG-3′	144
TGF-β	5′-AGGGCTACCATGCCAACTTC-3′	5′-CCACGTAGTAGACGATGGGC-3′	168
β-Catenin	5′-GCTGCACAGGTGACCACATT-3′	5′-GAGTGCTGAAGGTGCTGTCTGT-3′	238
Notch-1	5′-GGCACTTTCTGTGAGGAGGA-3′	5′-GCAGTCAGGCGTGTTGTTCT-3′	147
GAPDH	5′-AGCCACATCGCTCAGACA-3′	5′-GCCCAATACGACCAAATCC-3′	66

*ZEB*-*1* E-box binding homeobox-1, *TGF*-*β* transforming growth factor beta, *GAPDH* glyceraldehyde-3-phosphate dehydrogenase, *bp* base pair

### Western blotting

Cell lysates were prepared according to standard procedures [34]. Whole cell lysates (50 μg) were separated via 10% sodium dodecyl sulfate–polyacrylamide gel electrophoresis and transferred to polyvinylidene fluoride membrane for Western blotting. The membranes were incubated with primary antibodies against E-cadherin, Claudin-1, Vimentin, Snail, β-catenin, Notch-1, Notch-2, Notch-4, Akt, GSK-3β, p-GSK-3β, NF-κB p65, and β-actin overnight at 4 °C. The bands were visualized using a chemiluminescence kit (Thermo Scientific, Waltham, MA, USA) after incubation with the corresponding horseradish peroxidase-conjugated secondary antibodies (Cell Signaling Technology). β-Actin was used as an internal control to confirm the equal loading amount of whole cell lysates.

### Stable transfection of E-cadherin

PC3-TxR and DU145-TxR cells (2 × 10^5^/well) were seeded in 12-well plates and cultured overnight at 37 °C. The E-cadherin-expressing lentiviral vector and control vector were diluted in 0.2 mL (1 × 10^8^ transducing units/mL) complete medium containing 5 μg/mL polybrene (Sigma) and added to cells for a 24-h incubation at 37 °C. After the complete medium was replaced, cells were incubated for another 48 h. Then, the culture medium was changed to complete medium containing puromycin (5 µg/mL, Invitrogen), which was replaced every 2 days for approximately 2 weeks until all the non-transfected cells had died. The expression of E-cadherin gene and protein was evaluated using qPCR and Western blotting, respectively.

### Small interfering RNA transfection

To knockdown E-cadherin expression, PC3 and DU145 cells were transfected with E-cadherin-specific or control siRNA. PC3 and DU145 cells (4 × 10^5^/well) were plated in 6-well plates and then transfected with 20 nmol/L siRNA using Lipofectamine 2000 (Invitrogen) and cultured for 48 h. Then, total RNA and protein were extracted. The expression of E-cadherin gene and protein was evaluated using qPCR and Western blotting, respectively.

### Colony formation assay

Cells were seeded in 6-well plates at a density of 400 cells/well, cultured for approximately 10 days, washed with 1× PBS, fixed with 4% formaldehyde for 15 min, and stained using crystal violet (Beyotime, Shanghai, China) for 15 min. The colony containing 50 or more cells was counted. Three independent experiments were performed.

### Transwell assay

The cell migration and invasion potentials were evaluated using transwell assay [32]. In the migration assays, tumor cells (1 × 10^5^) were seeded into the upper chamber in RPMI-1640 medium without FBS. The lower chamber contained RPMI-1640 containing 10% FBS. In the invasion assays, growth factor-reduced (GFR) Matrigel invasion chambers (Becton–Dickinson, Franklin Lakes, NJ, USA) were used, and cells were seeded into the upper chamber of the transwell insert. After incubation for 24 h, the non-migrating or non-invading cells were gently removed using a cotton swab. The remaining cells were then fixed with 4% formaldehyde for 5 min, stained with crystal violet for 10 min, and counted in five fields under an inverted microscope. The independent experiments were repeated three times.

### Wound healing assay

Wound healing assays were used to evaluate cell migration. The cells (1  ×  10^6^/well) were seeded in 6-well plates. After the cells formed a confluent monolayer, scratches were made using a 200-μL pipette tip. The cells were then washed with 1× PBS to remove floating cells, and the wound closure was observed at indicated time points and photographed under a microscope. All the results were analyzed using the ImageJ software (National Institutes of Health, Bethesda, Maryland, USA). Wound repair (%) was calculated as following: Wound repair = [(Diameter of the wound before migration—Diameter of the wound after migration)/Diameter of the wound before migration] × 100%. The mean value of wound repair in three duplicate wells was calculated for each group.

### MTS assay

Cell survival was measured using a Cell Titer96^®^ Aqueous One Solution Cell Proliferation Assay (Promega, Madison, WI, USA) in the light of the manufacturer’s instruction. Briefly, cells were seeded in a 96-well plate (2000 cells/well) and incubated with paclitaxel for indicated time, then 20 μL MTS solution was added to each well and incubated with cells at 37 °C for another 2 h, at last the absorbance (*A*_490_) of each well was read at 490 nm. Cells without paclitaxel treatment were used as controls. The survival rate was calculated: Survival rate = (*A*_490_ of test cells/*A*_490_ of control cells) × 100%. The 50% inhibitory concentrations (IC_50_) were calculated.

### ONCOMINE database analysis of E-cadherin expression

The expression of E-cadherin in PCa tissues was assessed by performing a meta-analysis of the ONCOMINE database, a cancer gene microarray database. Multiple comparisons among different studies were analyzed on the basis of ONCOMINE algorithms to explain the differences in their results, similar to a meta-analysis, as previously described [35].

### Statistical analysis

Descriptive statistics including mean values and standard error of the mean (SEM) were calculated using Microsoft Excel and Prism software (GraphPad, Droitwich, Worcestershire, UK). All data were from at least three independent experiments; and one-way analysis of variance (ANOVA) and Student’s *t* tests were used to analyze the data.

## Results

### Chemoresistant PCa cells exhibited EMT morphologic changes and expressed EMT-associated markers

**Fig. 1:**
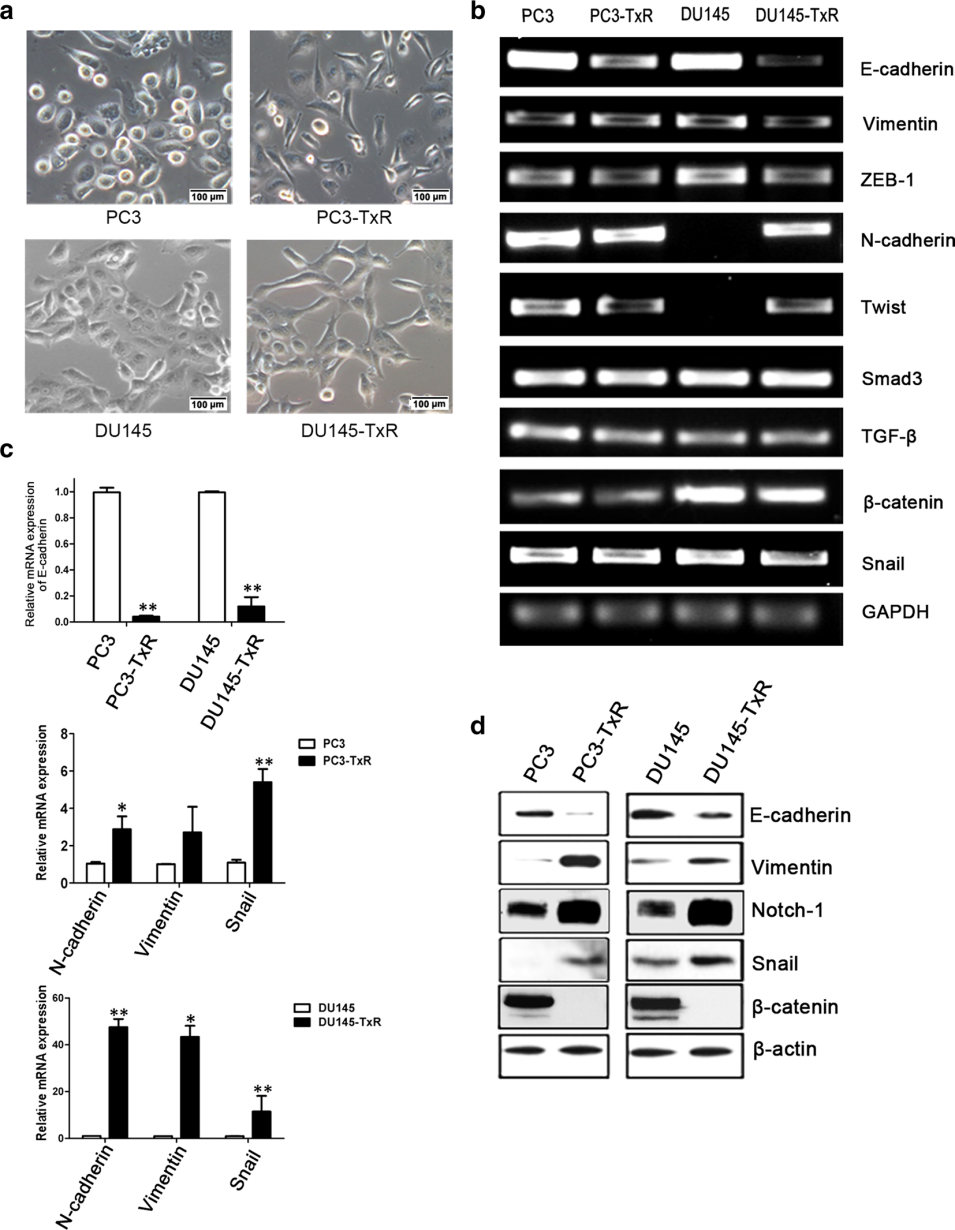
Chemoresistant prostate cancer (PCa) cells show epithelial-to-mesenchymal transition (EMT) changes comparing with their parental cells. **a** Morphology of parental PC3 and DU145 cells, and chemoresistant PC3-TxR and DU145-TxR cells was observed under a microscope at 200× magnification. **b** The expression of EMT markers in PC3, DU145, PC3-TxR, and DU145-TxR cells was detected using semi-quantitative reverse transcription-polymerase chain reaction (RT-PCR). *ZEB*-*1*, E-box binding homeobox-1; *TGF*-*β*, transforming growth factor beta; *GAPDH*, glyceraldehyde-3-phosphate dehydrogenase. **c** Quantitative real-time PCR (qPCR) analysis results show alterations in the mRNA levels of EMT markers in chemoresistant cells comparing with parental cells. **d** The expression of EMT-related proteins in PC3, DU145, PC3-TxR, and DU145-TxR cells was analyzed using Western blotting assay. Quantification data are presented as mean ± standard error of the mean (SEM). **P < 0.01, *P < 0.05

We first observed the morphologic changes in PC3-TxR and DU145-TxR cells compared with their parental PC3 and DU145 cells, respectively. PC3-TxR and DU145-TxR cells exhibited a spindle-shaped morphology and were dispersed, whereas PC3 and DU145 cells were round and assembled (Fig. 1a). Semi-quantitative RT-PCR, qPCR, and Western blotting results showed that, in PC3-TxR and DU145-TxR cells, the mRNA and protein levels of the epithelial marker E-cadherin were significantly reduced, whereas the levels of mesenchymal markers including Vimentin, Snail, and N-cadherin were increased compared with those in PC3 and DU145 cells, respectively (Fig. 1b–d).

### Chemoresistant PCa cells exhibited enhanced migratory and invasive abilities

**Fig. 2:**
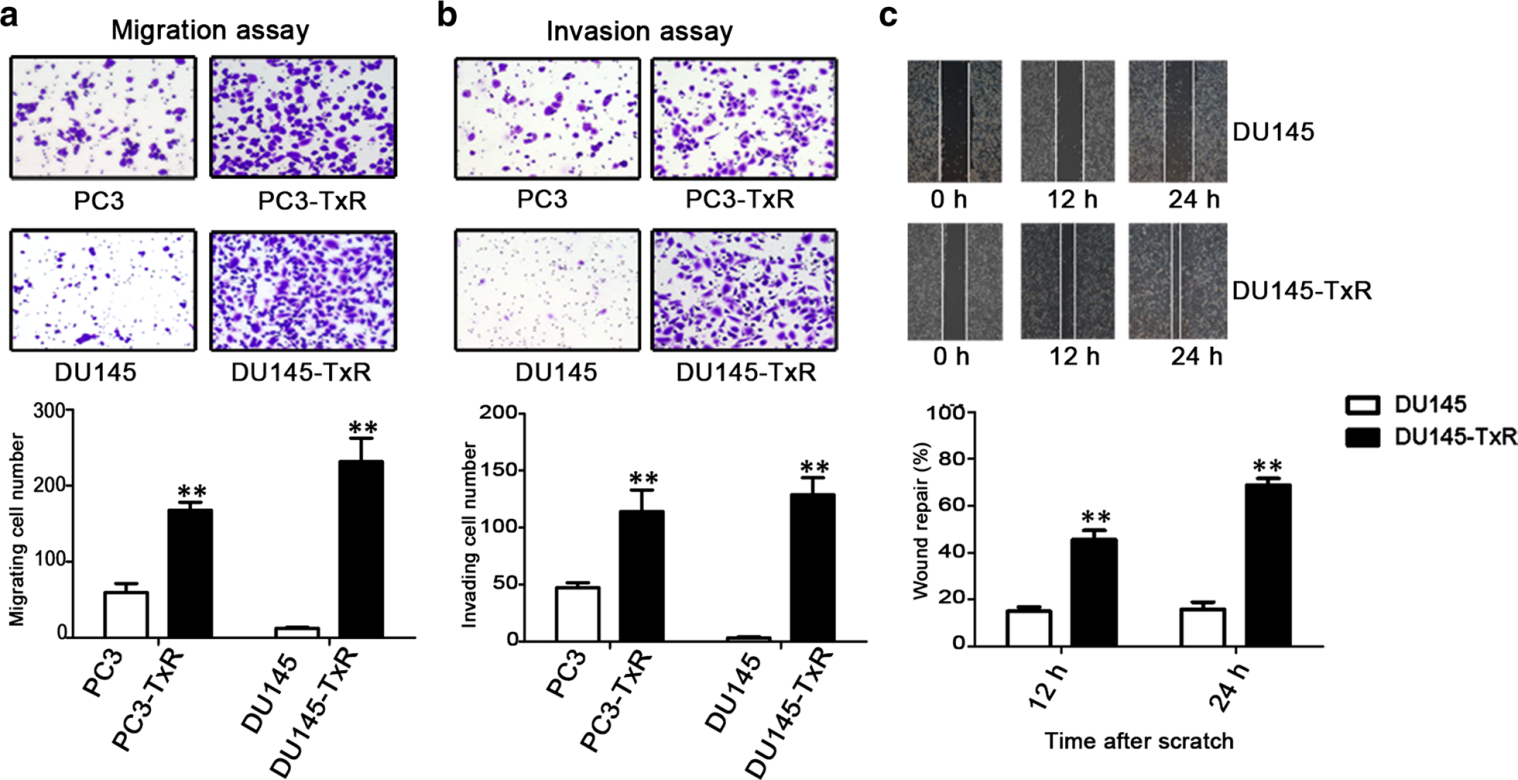
Chemoresistant PCa cells show enhanced migration and invasion abilities in vitro. **a** Migratory abilities of PC3, DU145, PC3-TxR, and DU145-TxR cells were determined using transwell assay. **b** Invasive abilities were determined using transwell assay. On each filter, five microscopic fields (at 200× magnification) were observed, and cells were counted. **c** Migratory abilities of DU145 and DU145-TxR cells were determined using wound healing assay. Wounded monolayers of DU145 and DU145-TxR cells were photographed 0, 12, and 24 h after the mechanical scratch, and the widths of the wounds were measured in 3-independent wound sites per group. Wound repair was calculated. The mean ± SEM values of data from three independent experiments are presented. *P < 0.05, **P < 0.01

Transwell assay results showed that the migratory and invasive abilities of PC3-TxR and DU145-TxR cells were significantly increased compared with PC3 and DU145 cells, respectively (Fig. 2a, b). Wound healing assay results showed that the migration of DU145-TxR cells was enhanced significantly compared with that of DU145 cells (Fig. 2c). The migratory ability of PC3-TxR cells was similarly enhanced as that of DU145-TxR cells (data not shown).

### Chemoresistant PCa cells grew faster than parental PCa cells in a xenograft mouse model

**Fig. 3:**
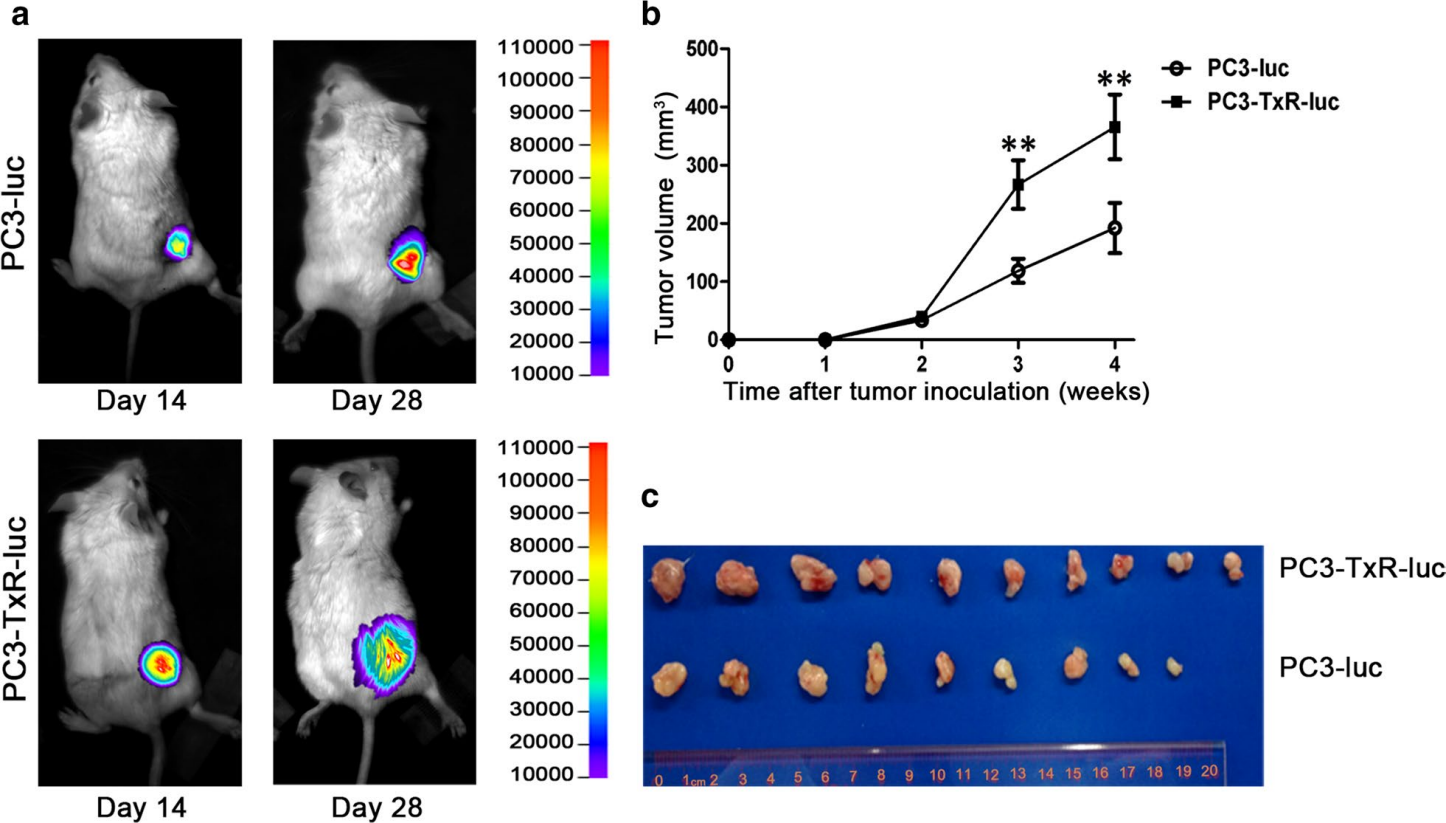
Chemoresistant PCa cells demonstrate enhanced subcutaneous tumor growth in mice. **a** Luminescence imaging of tumors in mice. PC3 and PC3-TxR cells were transfected with luciferase lentivial vector to construct PC3-luc and PC3-TxR-luc cells, respectively. PC3-luc and PC3-TxR-luc cells (4 × 10^6^ cells/mouse) were injected subcutaneously into the right back of male severe combined immune deficiency (SCID) mice, respectively; live images on days 14 and 28 are shown. **b** Tumor growth curves of PC3-luc and PC3-TxR-luc cell xenografts. Tumor volumes were recorded once a week. The mean ± SEM values of data from 10 mice per group are presented. **c** The photo of subcutaneous tumors excised from mice when the tumor volume was approximately 1 cm^3^. *P < 0.05, **P < 0.01

To assess the tumorigenesis of chemoresistant and parental PCa cells in vivo, PC3-TxR and PC3 cells that express luciferase, named PC3-TxR-luc and PC3-luc cells, respectively, were injected subcutaneously into SCID mice; tumor growth was monitored. As shown in Fig. 3a, the photon intensities in PC3-TxR-luc cell-implanted mice were significantly higher than those in the PC3-luc cell-implanted mice. The tumor growth curves and final tumor sizes showed that PC3-TxR-luc tumors grew faster than PC3-luc tumors in mice (Fig. 3b, c).

### E-cadherin overexpression inhibited PC3-TxR and DU145-TxR cell migration and invasion and partly restored paclitaxel sensitivity

**Fig. 4:**
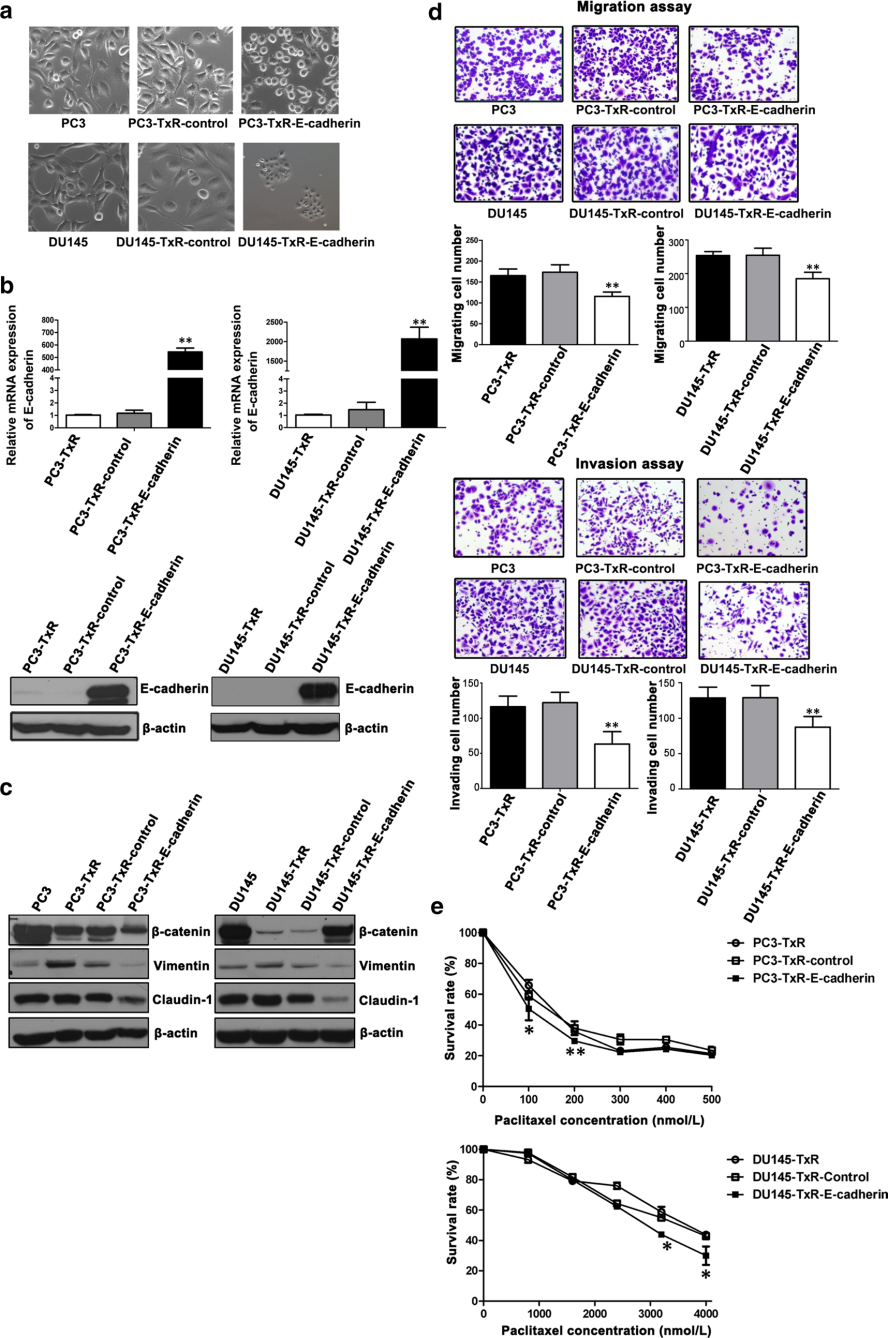
Overexpressing E-cadherin inhibits PC3-TxR and DU145-TxR cell migration and invasion and partly restores paclitaxel sensitivity. **a** PC3-TxR-E-cadherin and DU145-TxR-E-cadherin cells show epithelial morphology. Cells transfected with control lentiviral vectors are named PC3-TxR-control and DU145-TxR-control, respectively; and cells transfected with E-cadherin-expressing lentiviral vectors are named PC3-TxR-E-cadherin and DU145-TxR-E-cadherin, respectively. Cell morphology was observed under a microscope at 200× magnification. **b** qPCR and Western blotting analysis of E-cadherin expression. **c** Western blotting analysis of β-catenin, Vimentin, and Claudin-1 expression. **d** Migratory and invasive abilities were analyzed using transwell assays, and representative photomicrographs of migrating and invading cells were quantified, respectively. **e** E-cadherin overexpression partly reverses paclitaxel resistance in chemoresistant PCa cells. All cells were treated with paclitaxel for 72 h. Cell survival was determined using MTS assay. The mean ± SEM values of data from three independent experiments are presented. *P < 0.05, **P < 0.01

Since E-cadherin expression was decreased in chemoresistant cells, PC3-TxR and DU145-TxR cells were transfected with E-cadherin-specific or control lentiviral vectors. After selection, the cells stably overexpressing E-cadherin, named PC3-TxR-E-cadherin and DU145-TxR-E-cadherin cells, were obtained; the control cells were named PC3-TxR-control and DU145-TxR-control. PC3-TxR-E-cadherin cells became round and assembled compared with PC3-TxR-control cells (Fig. 4a). The transfection efficiency was measured using qPCR (Fig. 4b) and Western blotting (Fig. 4b), which confirmed the high expression of E-cadherin in PC3-TxR-E-cadherin cells. Western blotting results for EMT markers showed that the expression of Vimentin and Claudin-1 was decreased in PC3-TxR-E-cadherin cells (Fig. 4c). Transwell assay results showed that the overexpression of E-cadherin was significantly associated with inhibited migration (Fig. 4d) and invasion (Fig. 4d). Results of DU145-TxR cells are similar to those of PC3-TxR cells.

To study the role of E-cadherin in the chemoresistance of PC3-TxR and DU145-TxR cells, the survival rates of chemoresistant, control, and E-cadherin-overexpressing PCa cells treated with different concentrations of paclitaxel were assessed using MTS assay. The results showed that survival rate was decreased in E-cadherin-overexpressing cells compared with those of control and parental cells (Fig. 4e). The IC_50_ at 72 h was 146.81 ± 1.46 nmol/L for PC3-TxR cells, 139.13 ± 4.60 nmol/L for PC3-TxR-control cells, and 96.20 ± 15.03 nmol/L for PC3-TxR-E-cadherin cells; 3831.95 ± 65.69 nmol/L for DU145-TxR cells, 3725.45 ± 87.36 nmol/L for DU145-TxR-control cells, and 3022.10 ± 34.01 nmol/L for DU145-TxR-E-cadherin cells. Together, these observations suggest that overexpression of E-cadherin plays an important role in inhibiting migration and invasion and partially restores paclitaxel sensitivity of chemoresistant PCa cells.

### Silencing E-cadherin expression caused EMT-mediated paclitaxel resistance in parental PCa cells

**Fig. 5:**
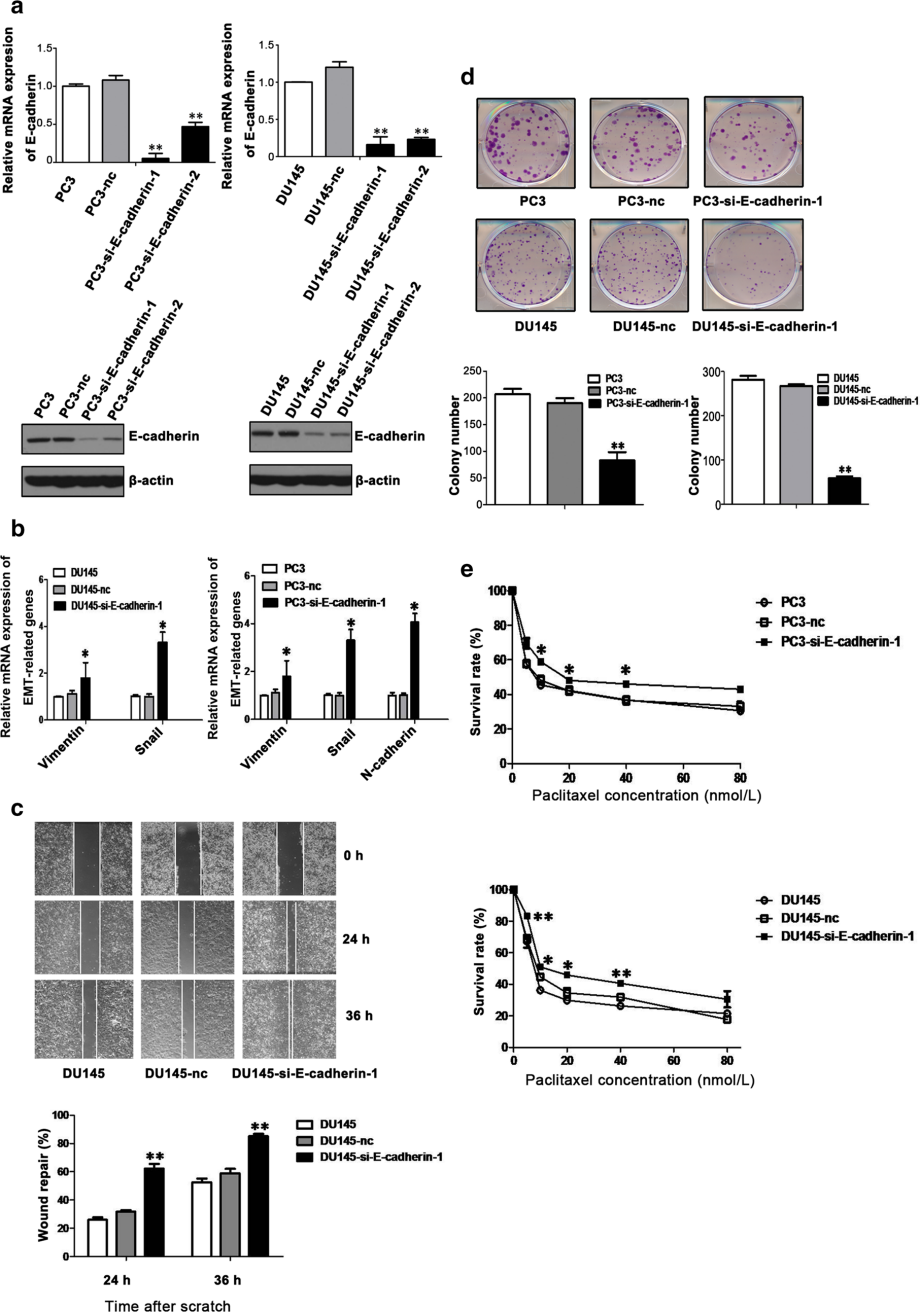
Silencing E-cadherin expression in PC3 and DU145 cells causes EMT-mediated paclitaxel tolerance. **a** qPCR (*top*) and Western blotting analysis (*bottom*) of E-cadherin mRNA and protein expression in PC3 and DU145 cell lines after small interfering RNA (siRNA)-mediated E-cadherin silencing. Cells transfected with negative control siRNA are named PC3-nc and DU145-nc, respectively; cells transfected with E-cadherin siRNA are named PC3-si-E-cadherin-1, PC3-si-E-cadherin-2, DU145-si-E-cadherin-1, and DU145-si-E-cadherin-2. **b** Expression of mesenchymal markers Snail, Vimentin, and N-cadherin were measured using qPCR. **c** Wounded DU145, DU145-nc, and DU145-si-E-cadherin-1 cell monolayers were photographed 0, 24, and 36 h after the mechanical scratch, and the width of the wounds was measured in 3 independent wound sites per group. **d** Colony formation abilities of parental cells, negative control cells, and E-cadherin-silencing cells were tested. The numbers of colonies are shown. **e** Silencing E-cadherin expression induces the resistance of PCa cells to paclitaxel. Cells were treated with paclitaxel (range: 0–80 nmol/L) for 72 h. Cell survival was determined using MTS assay. The mean ± SEM values of data from three independent experiments are presented. **P < 0.01, *P < 0.05

To further assess the role of E-cadherin in the chemoresistance of PCa cells, two different E-cadherin siRNAs were used to silence E-cadherin expression in PC3 and DU145 cells (si-E-cadherin-1, target sequence 2370–2389; si-E-cadherin-2, target sequence 800–818). The effective silencing of E-cadhering was confirmed by qPCR (Fig. 5a) and Western blotting (Fig. 5a). The expression of EMT markers such as Vimentin, Snail, and N-cadherin was up-regulated in PC3-si-E-cadherin-1 and DU145-si-E-cadherin-1 cells (Fig. 5b). Wound healing assay results showed that the migration of DU145-si-E-cadherin-1 cells was increased compared with that of control cells (Fig. 5c). Colony formation assays demonstrated that silencing E-cadherin in PC3 and DU145 cells inhibited colony formation (Fig. 5d). MTS assay results showed that cell survival rate was increased in E-cadherin-silencing cells compared with those of control and parental cells when treated with paclitaxel (Fig. 5e), with IC_50_ at 72 h being 9.49 ± 0.89 nmol/L for PC3 cells, 9.71 ± 2.38 nmol/L for PC3-nc cells, 14.73 ± 1.58 nmol/L for PC3-si-E-cadherin-1 cells, 8.31 ± 1.24 nmol/L for DU145 cells, 8.77 ± 2.40 nmol/L for DU145-nc cells, and 17.03 ± 1.54 nmol/L for DU145-si-E-cadherin-1 cells. The sensitivity to paclitaxel was decreased in E-cadherin-silenced PCa cells.

### Inhibiting the Notch pathway reversed the resistance to paclitaxel

**Fig. 6:**
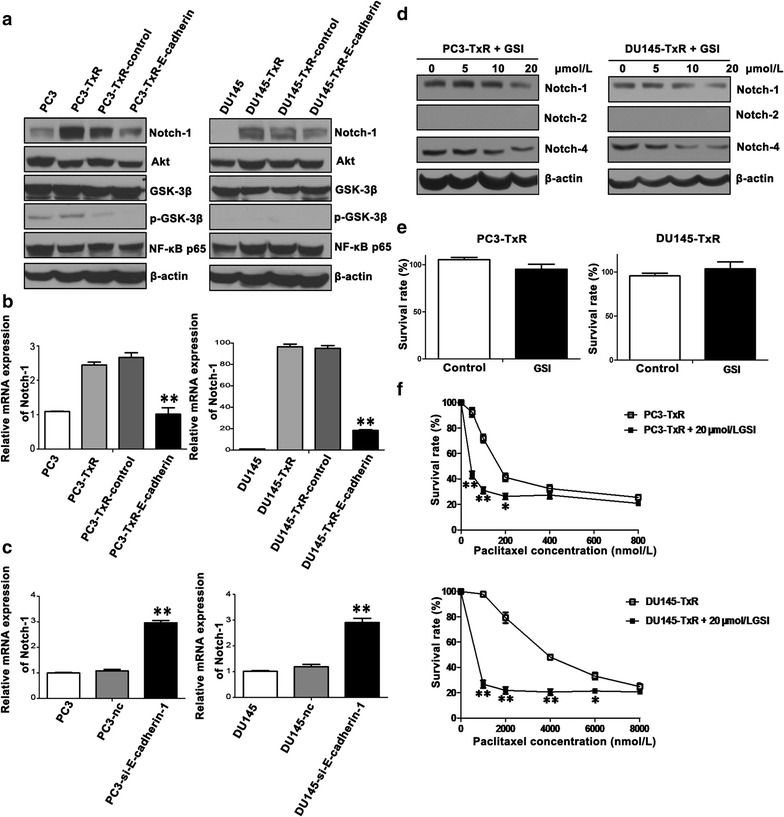
Gamma-secretase inhibitor (GSI) significantly increases the sensitivity of chemoresistant cells to paclitaxel. **a** Western blotting analysis of the expression of Notch-1, Akt, glycogen synthase kinase-3β (GSK-3β), phosphorylated GSK-3β (p-GSK-3β), nuclear factor kappa-light-chain-enhancer of activated B cells (NF-κB) p65, and β-actin in parental, chemoresistant, control vector-transfected, and E-cadherin-overexpressing PCa cells. **b** qPCR analysis of Notch-1 expression in the above mentioned cells. **c** qPCR analysis of Notch-1 expression in E-cadherin-silenced PCa cells. **d** Notch-1 expression is inhibited by GSI. Cells were treated with GSI (0, 5, 10, 20 µmol/L) for 72 h. The protein levels of Notch-1, Notch-2, and Notch-4 were assayed using Western blotting. **e** GSI does not inhibit the proliferation of PC3-TxR and DU145-TxR cells. Cells were treated with GSI (20 μmol/L), and cell survival was determined using MTS assay. **f** GSI reverses chemoresistance of PCa cells to paclitaxel. PC3-TxR and DU145-TxR cells were incubated with or without 20 µmol/L GSI in the presence of paclitaxel for 72 h. Cell survival was determined using MTS assay. The mean ± SEM values of data from 3 independent experiments are presented. *P < 0.05, **P < 0.01

To explore whether the Notch signaling pathway is involved in EMT-mediated chemoresistance, Notch protein levels of parental, chemoresistant, E-cadherin-overexpressing cells, and their vector control cells were measured. Western blotting results confirmed that the expression of Notch-1 was up-regulated in PC3-TxR and DU145-TxR cells and down-regulated in PC3-TxR-E-cadherin and DU145-TxR-E-cadherin cells (Fig. 6a). Similar results were observed using qPCR (Fig. 6b). Furthermore, Notch-1 levels were up-regulated in PC3-si-E-cadherin-1 and DU145-si-E-cadherin-1 cells (Fig. 6c). Next, PC3-TxR and DU145-TxR cells were treated with GSI, a Notch inhibitor, for 72 h, which inhibited the expression of Notch-1 and Notch-4 in both cell lines (Fig. 6d). GSI (20 µmol/L) alone did not affect the proliferation of PC3-TxR and DU145-TxR cells (Fig. 6e). However, GSI treatment significantly restored the sensitivity of chemoresistant cells to paclitaxel (Fig. 6f). The 72-h IC_50_ of paclitaxel for cells treated with GSI and paclitaxel was 13.90 ± 1.59 nmol/L for PC3-TxR cells and 838.00 ± 134.40 nmol/L for DU145-TxR cells, which was reduced by 90.5% and 78.8% compared with that for PC3-TxR and DU145-TxR cells treated with paclitaxel alone, respectively.

### E-cadherin expression significantly decreased in PCa clinical tissues

**Fig. 7:**
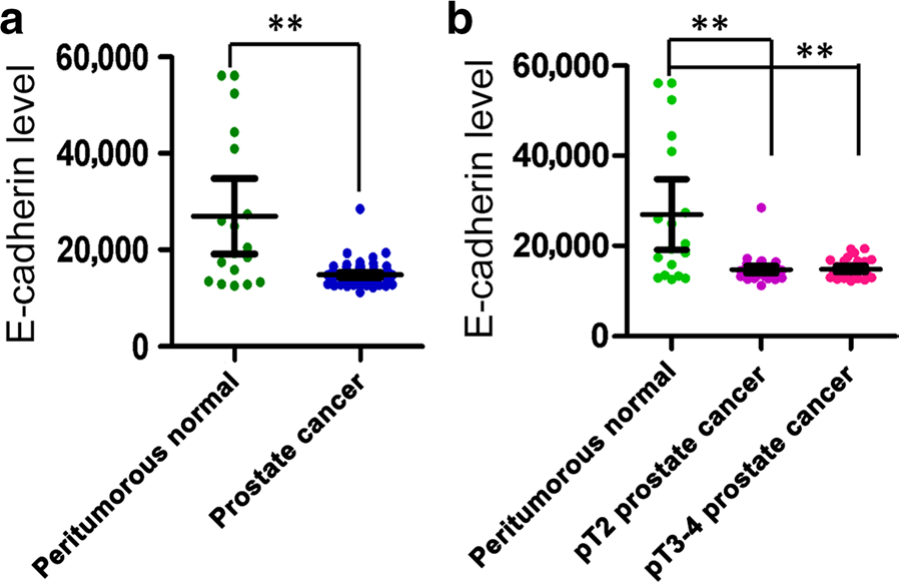
E-cadherin mRNA expression level decreases in PCa tumors in the meta-analysis of the ONCOMINE database. **a** E-cadherin mRNA expression level is lower in PCa tissues than in peritumorous normal prostate tissues. **b** E-cadherin mRNA expression is significantly lower in pT2 and pT3-4 PCa tumors than in peritumorous normal prostate tissues. The *P* value was calculated by Student’s *t* test, **P < 0.01, *P < 0.05

Finally, we detected E-cadherin mRNA expression in clinical tissue specimens by analyzing gene expression datasets in the ONCOMINE database. The data of E-cadherin expression in 69 prostate tumor tissues (inclcuding 38 pT2 cases and 31 pT3-4 cases) and 18 peritumorous normal prostate tissues detected using Affymetrix HG-U133A 2.0 microarrays [35] were collected. The results showed that E-cadherin expression was significantly decreased in PCa tissues compared with that in peritumorous normal tissues (Fig. 7).

## Discussion

The resistance to taxanes (paclitaxel or docetaxel) may account for tumor relapse and progression, which results in skeletal metastasis and high mortality [36]. Nevertheless, it is undefined how PCa progresses and specifically how chemoresistance occurs. In this study, we observed that the morphologic and functional characteristics differed in chemoresistant and parental PCa cells. EMT-associated markers were also evaluated, and results showed that the expression of E-cadherin was down-regulated and the expression of Vimentin, Snail, and N-cadherin was up-regulated in chemoresistant cells compared with parental PCa cells. Enhanced migration and invasion were also observed in chemoresistant cells using transwell assays. In addition, chemoresistant cells grew faster in male SCID mice than their parental PCa cells. Together, these observations strongly suggest that EMT is closely associated with chemoresistance in PCa cells.

E-cadherin is a classical cell–cell junction protein that is considered a hallmark of EMT [37–39]. In the current study, the overexpression of E-cadherin in chemoresistant PCa cells resulted in the down-regulation of Vimentin and Claudin-1 and inhibited cell migration and invasion. Next, the results of sensitivity to paclitaxel demonstrated that the overexpression of E-cadherin may reverse the chemoresistance in PCa. Meanwhile, silencing E-cadherin expression in parental cells increased cell migration, inhibited colony formation, and enhanced paclitaxel resistance of PCa cells. Together with the data of E-cadherin expression from the ONCOMINE database, all the results indicated that down-regulation of E-cadherin expression might play a fundamental role in PCa chemoresistance and metastasis.

To further understand the mechanism behind EMT-mediated chemoresistance, EMT-related signaling pathways involving Akt, GSK-3β, Notch, and NF-κB [37–39] were tested. The Notch signaling pathway was identified to be altered in the current study. The up-regulation of components of the Notch pathway has been observed in clinical samples of PCa [40], suggesting that the Notch pathway may play a crucial role in PCa progression. A correlation between E-cadherin and Notch-1 has been reported in trophoblast cells [38] and pancreatic cancer cells [41]. However, the relationship between the Notch signaling pathway and E-cadherin-mediated chemoresistance in PCa is unclear. In the current study, changes of Notch-1 expression were shown to be opposite to that of E-cadherin expression in both chemoresistant and parental PCa cells. An important characteristic of the Notch pathway is that all ligands and receptors are type I membrane proteins [42]. After cell-to-cell interactions, γ-secretase proteolytically cleaves Notch receptors to release a smaller transcriptional transactivator of Notch, Notch intracellular domain (NICD), which translocates into the nucleus to modulate the expression of down-stream genes [43]. Therefore, we tested the effect of GSI on PCa cells. The results showed that GSI inhibited the expression of Notch-1 and Notch-4 in chemoresistant PCa cells. Further studies showed that GSI could remarkably increase the sensitivity of chemoresistant cells to paclitaxel. These results suggest that down-regulation of E-cadherin contributes to PCa chemoresistance via the Notch signaling pathway.

## Conclusions

The current study demonstrated that down-regulation of E-cadherin contributes to EMT-mediated chemoresistance of PCa. E-cadherin could be a key and “driver” of the EMT morphologic changes, and its regulation might dictate PCa cell migration and invasion in vitro as well as tumor growth in vivo. Importantly, modulating Notch signaling appears to be important since the Notch inhibitor GSI significantly increased the sensitivity of PCa cells to paclitaxel. Further pre-clinical testing of this combination therapy may provide a promising novel strategy for PCa treatment.

## Abbreviations

PCa: prostate cancer
EMT: epithelial-to-mesenchymal transition
siRNA: small interfering RNA
SCID: severe combined immune deficiency
GSK-3β: glycogen synthase kinase 3 beta
NF-κB: nuclear factor kappa-light-chain-enhancer of activated B cells
NICD: Notch intracellular domain
ZEB-1: E-box binding homeobox-1
TGF-β: transforming growth factor beta
GAPDH: glyceraldehyde-3-phosphate dehydrogenase
